# Standard b-value DWI-derived stiffness index analysis may provide a way to evaluate the development of intracerebral hematoma

**DOI:** 10.3389/fneur.2024.1527861

**Published:** 2025-01-29

**Authors:** Qian Li, Jin Mao, Qiyuan Wang, Liding Yao, Fangfang Xu, Fei Dong

**Affiliations:** Department of Radiology, The Second Affiliated Hospital, Zhejiang University School of Medicine, Hangzhou, China

**Keywords:** elasticity imaging techniques, shear modulus, diffusion magnetic resonance imaging, cerebral hemorrhage, stiffness index, magnetic resonance elastography

## Abstract

**Background and purpose:**

The development of intracerebral hemorrhage (ICH) is closely related to mechanical forces. However, noninvasively evaluating mechanical forces for ICH patients in the current clinical setting is challenging. In this study, we aimed to build an easily accessible stiffness index (STI) and evaluate the stiffness of the perihematomal edema (PHE) region in ICH patients.

**Materials and methods:**

In this retrospective study, two cohorts of 57 patients were included. One cohort (the exploratory cohort) comprised patients with both standard b-value diffusion-weighted imaging (sDWI) (b-values of 0 and 1,000 s/mm^2^, b0 and b1000) and higher b-value diffusion-weighted imaging (hDWI) (b-values of 200 and 1,500 s/mm^2^). Another cohort (the hemorrhage cohort) consisted of patients who were diagnosed with ICH and who underwent sDWI within 48 h from onset. The hDWI-based virtual shear modulus (μdiff) was calculated and correlated with the sDWI data in the exploratory cohort. In the hemorrhage cohort, STI maps that were used to estimate μdiff were generated. The mean STI (mSTI) and coefficient of variation (COV) of the STI were computed on the basis of the STI maps in the whole and largest-slice PHE regions.

**Results:**

The STI could be calculated with the Equation 0.047697*S1000-0.022944*S0 + 5.359883, where S1000 and S0 represent the signal intensities of the b1000 and b0 images, respectively. In the whole and largest-slice PHE regions, both the mSTI and COV were correlated with the hematoma volume (*p* < 0.01), but neither were correlated with the time from onset.

**Conclusion:**

The standard b-value DWI-derived stiffness index analysis may provide a noninvasive and easily accessible way to evaluate the development of ICH.

## 1 Introduction

Intracerebral hemorrhage (ICH) is a devastating form of stroke (1), with 3.41 million cases occurring each year worldwide, accounting for 27.9% of all stroke patients (2). The median fatality was 40·4% at 1 month and 54·7% after 1 year (3).

ICH is typically divided into five stages based on the breakdown products of blood, including hyperacute (<12 h), acute (12 h–48 h), early subacute (2 days–7 days), late subacute (8 days–1 month), and chronic (>1 month) stages (4). The first 48 h are very important period. During this period of ICH development, it involves hematoma expansion (4, 5), the occurrence of perihematomal edema (6), primary and secondary brain injury (5), and is correlated with high mortality (7).

It has been reported that the development of ICH is closely related to mechanical force (8–10), and the perihematomal area serves as a critical interface for both deleterious and beneficial effects (8). Furthermore, stiffness reflects the ability of a structure to resist deformation under force (11). Therefore, measuring the stiffness of the perihematomal area may be valuable for evaluating the development of incipient ICH from a mechanical viewpoint and may provide a target for monitoring and treatment for ICH patients.

Although magnetic resonance elastography (MRE) provides a quantitative method for evaluating tissue stiffness and has been used in various central nervous system diseases (12), it is necessary to consider that there may be a potential risk of exacerbating the disease due to mechanical vibrations during the examination. Currently, MRE has not been found to be used for ICH patients.

Recently, diffusion-weighted (DW) MRI-based elastography or virtual MRE (vMRE) has been proposed. This method involves fitting the shear modulus with DWI parameters to avoid the need for mechanical vibration. It has demonstrated excellent agreement with MRE in staging liver fibrosis (13), and has shown consistent results compared to surgery in cases of meningiomas and pituitary adenomas (14, 15). In these studies, higher b-value (200 s/mm^2^ and 1,500 s/mm^2^, or 200 s/mm^2^ and 1,000 s/mm^2^) were used. However, standard b-value (0 s/mm^2^ and 1,000 s/mm^2^) DWI is more commonly used in clinical settings.

In this study, we aimed to build an easily accessible standard b-value DWI-derived stiffness index (STI) and evaluate the stiffness of the perihematomal edema (PHE) region, which is an important region of the perihematomal area (16), in ICH patients within 48 h from onset.

## 2 Materials and methods

The retrospective study was approved by the Local Ethics Committee of our hospital, and the requirement for patient informed consent was waived.

### 2.1 Study population

We retrospectively included two cohorts (the exploratory cohort and hemorrhage cohort) of adult patients who underwent brain MRI examination at our hospital between January 2020 and October 2023. Patients in the exploratory cohort, including patients with both standard b-value DWI (sDWI, with b-values of 0 and 1,000 s/mm^2^, b0 and b1000 images) and higher b-value DWI (hDWI, with b-values of 200 and 1,500 s/mm^2^, b200 and b1500 images), were used to investigate the relationships between sDWI parameters and virtual shear modulus. Patients in the hemorrhage cohort, including ICH patients with sDWI within 48 h from onset, were analyzed to assess the stiffness (the magnitude of the shear modulus is sometimes called stiffness, a term that is intuitive to both the general public and clinicians (17)) of the PHE region. For the exploratory cohort, all patients underwent MRI examinations due to symptoms such as discomfort, headaches, and dizziness, and patients with significant intracranial diseases on MR images were excluded. For the hemorrhage cohort, patients who had undergone surgical intervention or who had ICH secondary to brain trauma, vascular malformation, brain tumor, and hemorrhagic infarction were excluded (18). The age and sex for both cohorts, as well as the baseline blood pressure and time from onset to MRI scan of each patient in the hemorrhage cohort, were recorded.

### 2.2 Image acquisition

For the exploratory cohort cases, MRI was performed using a 1.5 T MAGNETOM Altea scanner (Siemens Medical Solutions) and a 3.0T uMR 790 scanner (United Imaging). DWI was acquired with the following parameters: repetition time (TR), 3,740–4,510 ms; echo time (TE), 79.26 or 84.26 ms; section thickness, 6.0 mm; b-values, 0 and 1,000 s/mm^2^ or 200 and 1,500 s/mm^2^ for the MAGNETOM Altea scanner. And TR, 2600 ms; TE, 84.6 ms; section thickness, 5.5 mm; b-values, 0 and 1,000 s/mm^2^ or 200 and 1,500 s/mm^2^ for the uMR 790 scanner. For the hemorrhage cohort cases, MRI was performed with 1.5 T Aera scanners (Siemens Medical Solutions). The DWI was acquired with the following parameters: TR, 3900–5,300 ms; TE, 104–115 ms; section thickness, 6 mm; b-value, 0 and 1,000 s/mm^2^.

### 2.3 Image preprocessing and segmentation

A series of image preprocessing steps was carried out to minimize discrepancies caused by variations in MR image acquisition conditions. Utilizing b0 images as reference images, registration was conducted for the other images, and eddy current correction was applied to all images using FSL^1^. Gibbs ringing artifacts were removed using MRtrix3^2^. N4 bias field correction and denoising was performed using CaPTK^3^ (19, 20).

Manual segmentation for all the cases was performed by two radiologists on b0 images using ITK-SNAP software^4^. For the exploratory cohort, 10 regions of interest (ROIs, 5 mm × 5 mm × 5 mm) were delineated on b0 images for each patient. The ROIs were chosen in the centrum semiovale, periventricular white matter, basal ganglia, thalamus, and cerebellar hemisphere on both sides. For the hemorrhage cohort, four ROIs were delineated, including the whole hematoma region, the whole PHE region, the largest-slice hematoma region and the largest-slice PHE region. The whole hematoma and whole PHE regions were delineated slice by slice for all the hematoma and PHE lesions, respectively. The largest-slice hematoma and the largest-slice PHE regions were delineated in the hematoma and edema regions, respectively, on the slice with the largest hematoma size that was evaluated by the two doctors. Ten patients in the hemorrhage cohort were randomly selected to evaluate the consistency of the segmentation performed by the two doctors, with the segmented regions including the whole PHE region and the largest-slice PHE region.

The mean signal intensity of b0 images, b1000 images, b200 images, and b1500 images (S0, S1000, S200, and S1500) in each ROI for the exploratory cohort cases was computed using Python software^5^. The apparent diffusion coefficient (ADC) value for standard DWI was calculated by the following formula: ADC = ln (S0/S1000)/1,000 (21). The virtual shear modulus (μdiff) was calculated by the following formula: μdiff = −9.8 ln (S200/S1500) + 14 (21).

For the exploratory cohort cases, four parameters derived from sDWI, including S0, S1000, S0/S1000, and ADC value were used to build a model (μModel) to predict μdiff. The entire exploratory cohort dataset was randomly split into training (70%) and test (30%) datasets. For the hemorrhage cohort, according to the algorithm of the μModel, virtual magnetic resonance elastography (STI map) was generated using sDWI parameters and FSL software. The signal intensity value was extracted by Python software from the ROIs of the STI maps. The mean STI (mSTI) and coefficient of variation (COV, standard deviation/mSTI) as the STI parameters in the whole PHE region and the largest-slice PHE region were recorded and represented by the whole mSTI, whole COV, largest-slice mSTI, and largest-slice COV, respectively.

### 2.4 Statistical analysis

The continuous variables were presented as the mean or medians, according to whether or not a normal distribution was present. Categorical data were presented as proportions. Univariate linear regression was performed to select parameters derived from sDWI for the exploratory cohort cases. Multiple linear regression was then used to evaluate the independent association of the selected parameters with μdiff. The correlation between two continuous variables was assessed using either Pearson or Spearman correlation methods and linear regression analysis. The Dice coefficient was used to evaluate the consistency of segmentation performed by the two doctors. The statistical significance levels were two-sided, with the statistical significance level set at 0.05. The statistical analyses were performed using the R software (version 4.4.1).^6^

## 3 Results

### 3.1 Participant characteristics

**Figure 1:**
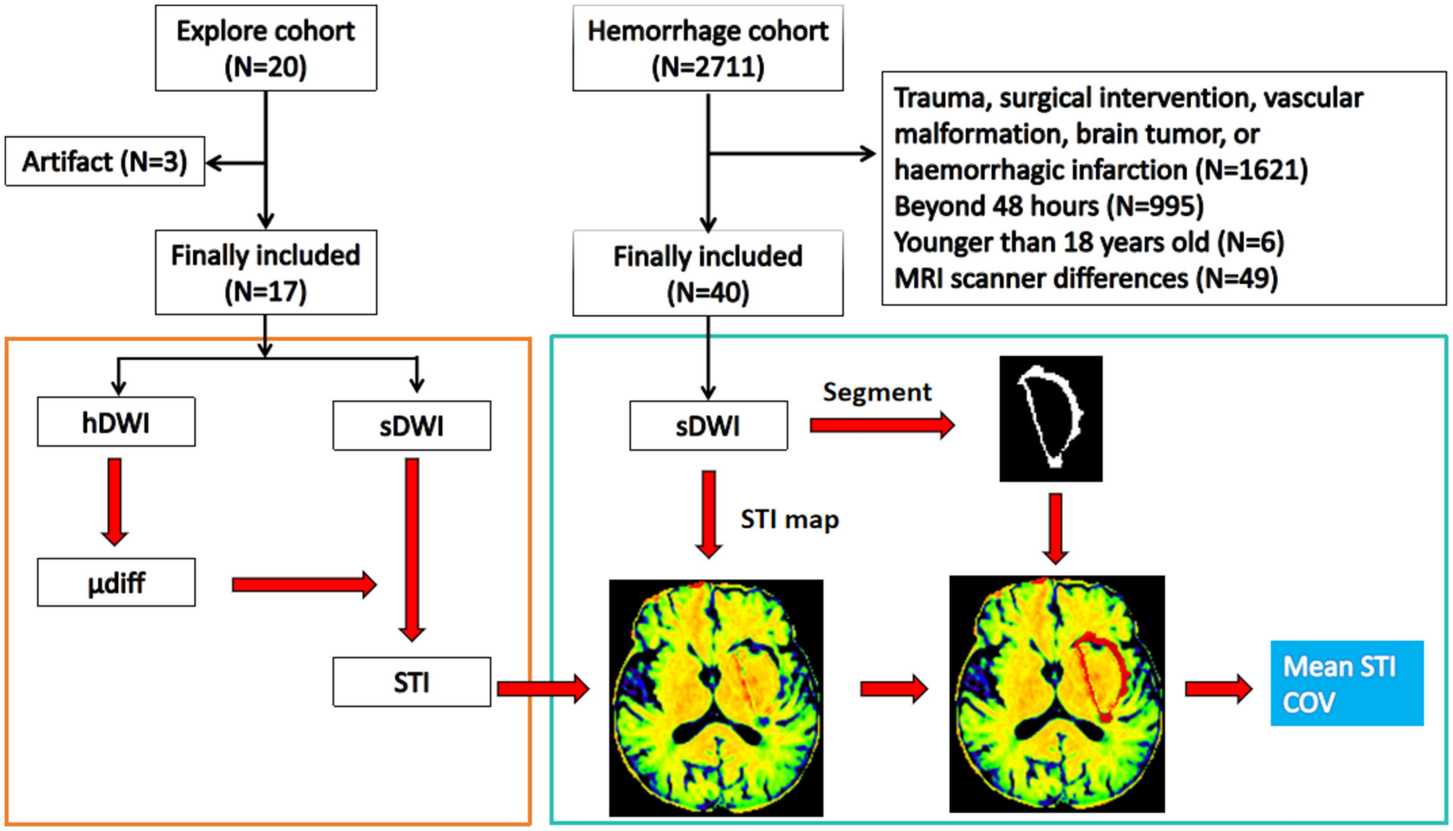
Flow diagram. The exploratory cohort is used to build the stiffness predicting model using standard b-value DWI, and the hemorrhage cohort is used to study the stiffness features of the perihematomal edema region. hDWI, high b-value diffusion weighted imaging; sDWI, standard b-value diffusion weighted imaging; STI, stiffness index; μdiff, diffusion-based virtual shear modulus; COV, coefficient of variation.

Fifty-seven patients, 32 (56.1%) men and 25 (43.9%) women, were included in this study. In the exploratory cohort, there were 17 patients, including 8 (47.1%) men and 9 (52.9%) women, and the age was 52.8 ± 14.8 years. In the hemorrhage cohort, there were 40 patients (Table 1 and Figure 1).

**Table 1:** Patient characteristics, hematoma and edema volumes, and STI features.

Characteristics	Values
Age, years	
Mean (sd)	57.1 (15.0)
Gender, *n* (%)	
Male, female	24 (60.0%), 16 (40.0%)
Time, hours	
Mean (sd)	24.6 (12.8)
SBP, mmHg	
Median (range)	150.5 (113.0–235.0)
DBP, mmHg	
Mean (sd)	84.5 (61.0–129.0)
Whole hematoma volume, mm^3^	
Median (range)	6562.9 (897.6–82759.5)
Largest-slice hematoma volume, mm^3^	
Median (range)	2643.6 (344.3–12408.8)
Whole edema volume, mm^3^	
Median (range)	7100.9 (609.6–55348.7)
Largest-slice edema volume, mm^3^	
Median (range)	2259.6 (475.3–7273.8)
Whole mSTI	
Mean (sd)	3.50 (1.80)
Largest-slice mSTI	
Mean (sd)	3.64 (2.06)
Whole COV	
Median (range)	0.56 (−4.15–3.74)
largest-slice COV	
Median (range)	0.45 (−5.88–9.54)

SBP, systolic blood pressure; DBP, diastolic blood pressure; sd, standard deviation; STI, stiffness index; mSTI, mean stiffness index; COV, coefficient of variation.

### 3.2 μModel building and validation

**Figure 2:**
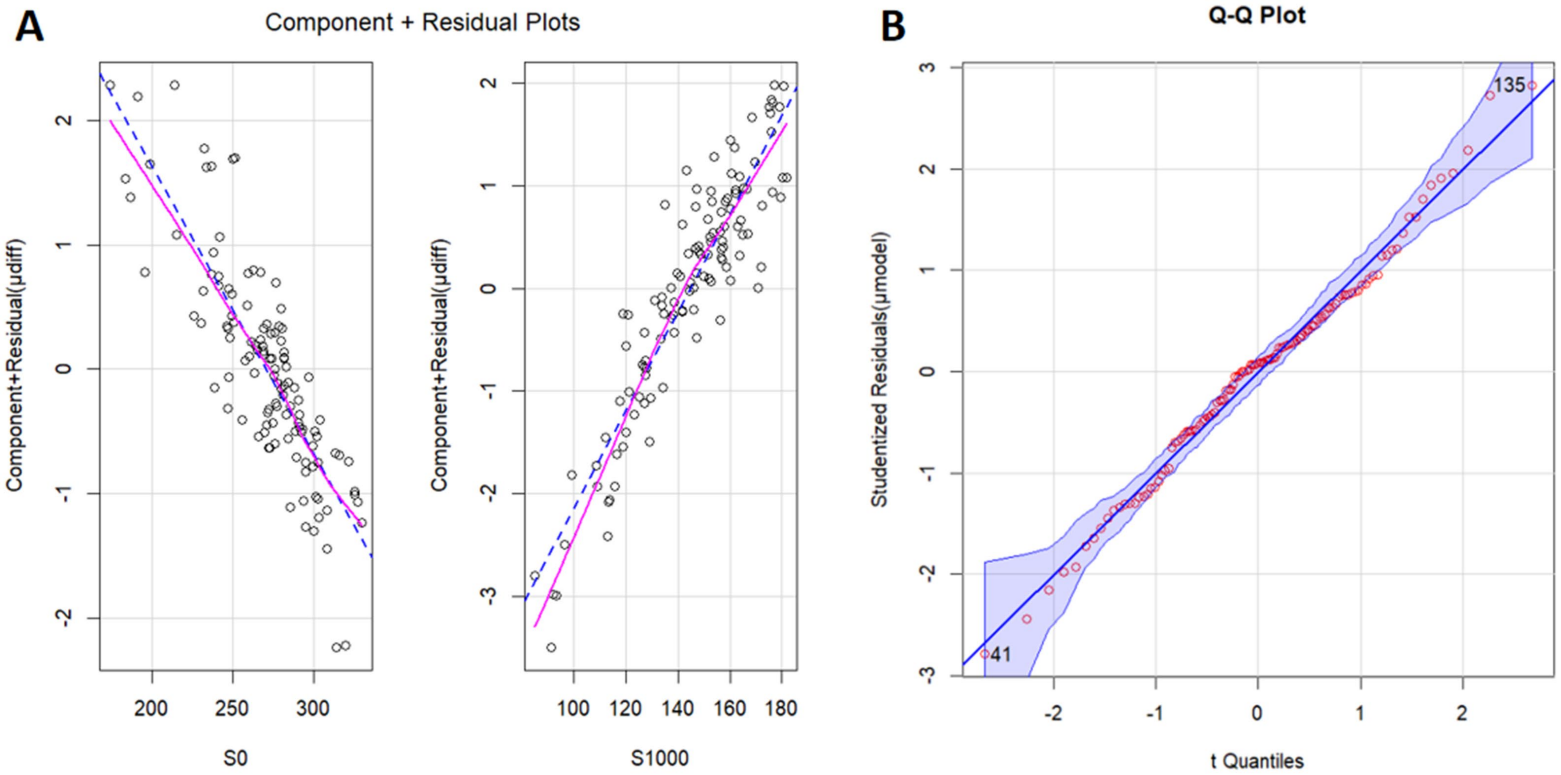
Component plus residual plot **(A)** and Q–Q plot **(B)**. The relationships between S0 and μdiff and between S1000 and μdiff are linear. S0, the signal intensity of b0 image (diffusion weighted imaging, *b* = 0 s/mm^2^); S1000, the signal intensity of b1000 image (diffusion weighted imaging, *b* = 1,000 s/mm^2^); μdiff, virtual shear modulus computed by diffusion weighted imaging using *b* = 200 and 1,500 s/mm^2^; μModel, model to predicting μdiff.

**Figure 3:**
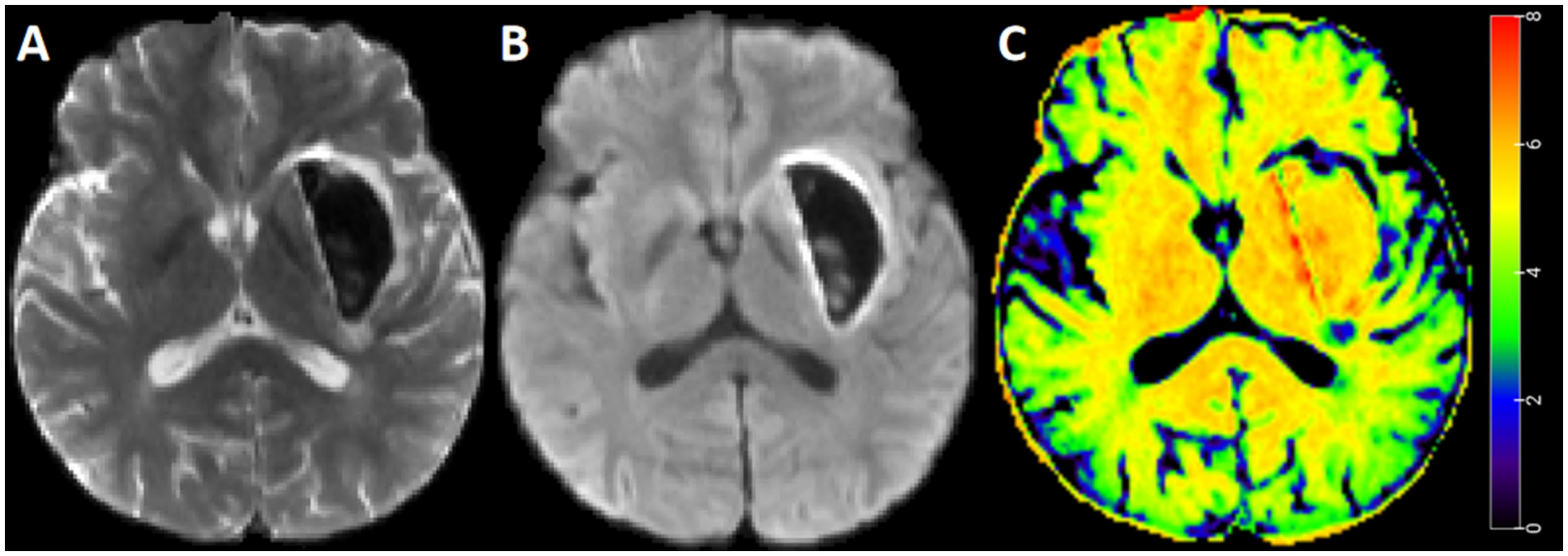
Standard b-value DWI and STI map for representative case. A 45-year-old man with intracerebral hemorrhage. Diffusion weighted imaging with *b* = 0 s/mm^2^ **(A)**, diffusion weighted imaging with *b* = 1,000 s/mm^2^ **(B)**, and STI map **(C)**. The STI in the perihematomal edema region was heterogeneous, with a relatively greater value in the deep region.

Univariate logistic regression revealed that each parameter, including S0 (*p* = 0.038), S1000 (*p* < 0.001), ADC (*p* < 0.001), and S0/S1000 (*p* < 0.001) contributed significantly to the prediction of μdiff. However, multivariable logistic regression showed that only S0 (*p* = 0.036) and S1000 (*p* = 0.043) were independent predictors (Figure 2). Using the model, the STI could be calculated with the Equation 0.047697*S1000-0.022944*S0 + 5.359883 (Figure 3), where S1000 and S0 represented the mean signal intensity of the ROIs on the b1000 and b0 images, respectively. The model showed an adjusted R-squared of 0.643 for the training dataset and 0.626 for the test dataset. The variance inflation factor was 3.04, which means that there was no serious multicollinearity between the two parameters.

### 3.3 Segmentation of the PHE region

The median Dice coefficient was 0.79 (range, 0.74–0.89) for the segmentation by the two doctors of the whole PHE region and 0.80 (range, 0.71–0.88) for the largest-slice PHE region, respectively. The median volumes of the whole PHE region and the largest-slice PHE region were 7100.9 mm^3^ and 2259.6 mm^3^, respectively (Table 1).

### 3.4 Evaluation of the STI

**Figure 4:**
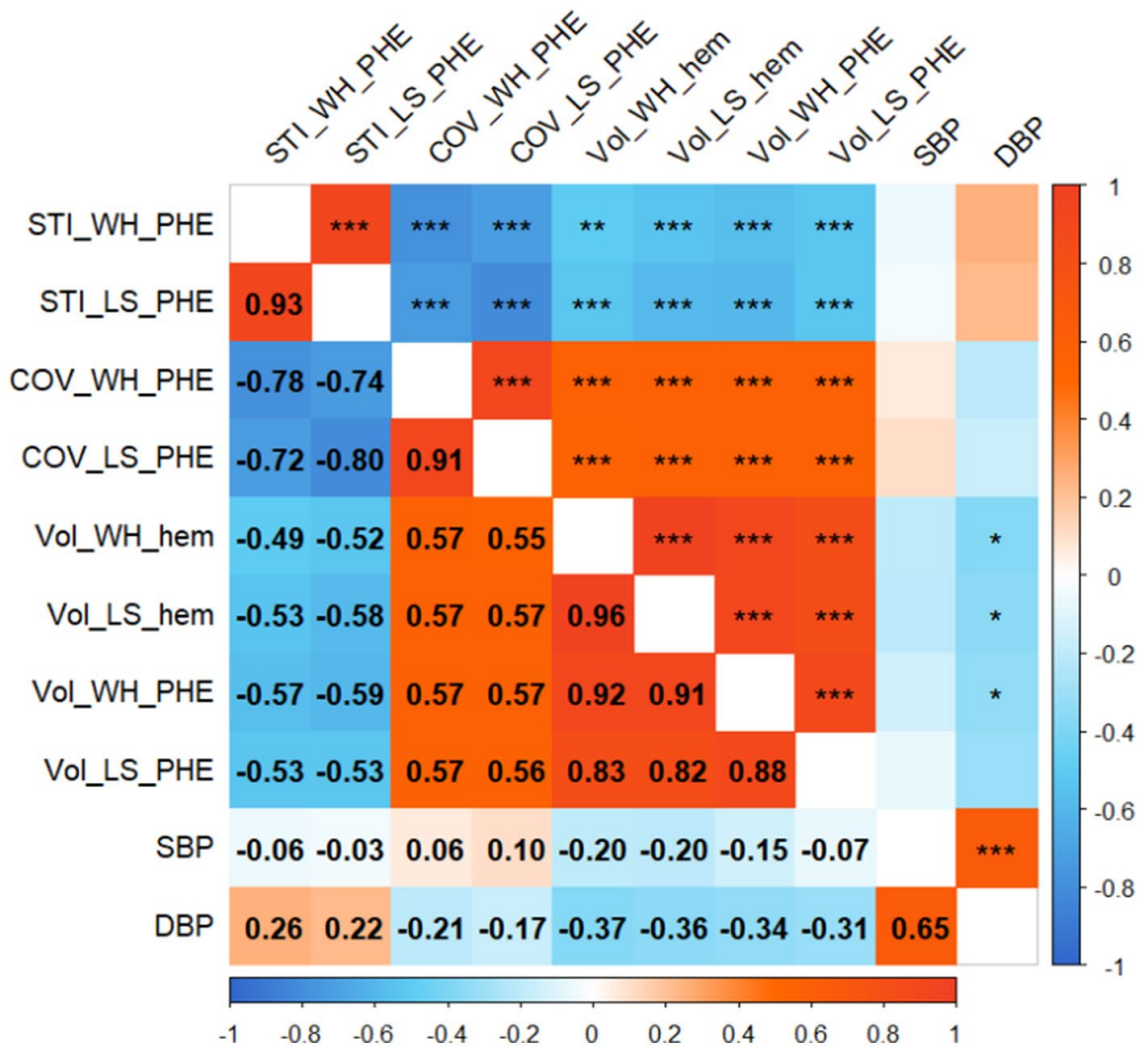
Correlogram. The numbers in the boxes indicate the correlation coefficient between the two variables. Significance is indicated by single asterisks (*p* < 0.05), double asterisks (*p* < 0.01), or triple asterisks (*p* < 0.001). STI_WH_PHE, mean stiffness index in the whole perihematomal edema region; STI_LS_PHE, mean stiffness index in the largest-slice perihematomal edema region; COV_WH_PHE, coefficient of variation of stiffness index in the whole perihematomal edema region; COV_LS_PHE, coefficient of variation of stiffness index in the largest-slice perihematomal edema region; Vol_WH_hem, the whole volume of hematoma; Vol_LS_hem, the largest-slice volume of hematoma; Vol_WH_PHE, the whole volume of perihematomal edema region; Vol_LS_PHE, the largest-slice volume of perihematomal edema region; DBP, diastolic blood pressure; SBP, systolic blood pressure.

The mSTIs exhibited a normal distribution, whereas the COVs exhibited a nonnormal distribution (Table 1). Overall, the largest-slice mSTI was positively correlated with the whole mSTI (*r* = 0.93), and the largest-slice COV was positively correlated with the whole COV (*r* = 0.91). Both the whole and the largest-slice mSTIs were negatively correlated with the largest-slice COV and with the whole COV (Figure 4).

Both the whole and largest-slice mSTIs were negatively correlated with the hematoma volume and PHE volume (*p* values, <0.01). However, both the whole COV and the largest-slice COV were positively correlated with the volumes (*p* values, <0.001) (Figure 4).

**Figure 5:**
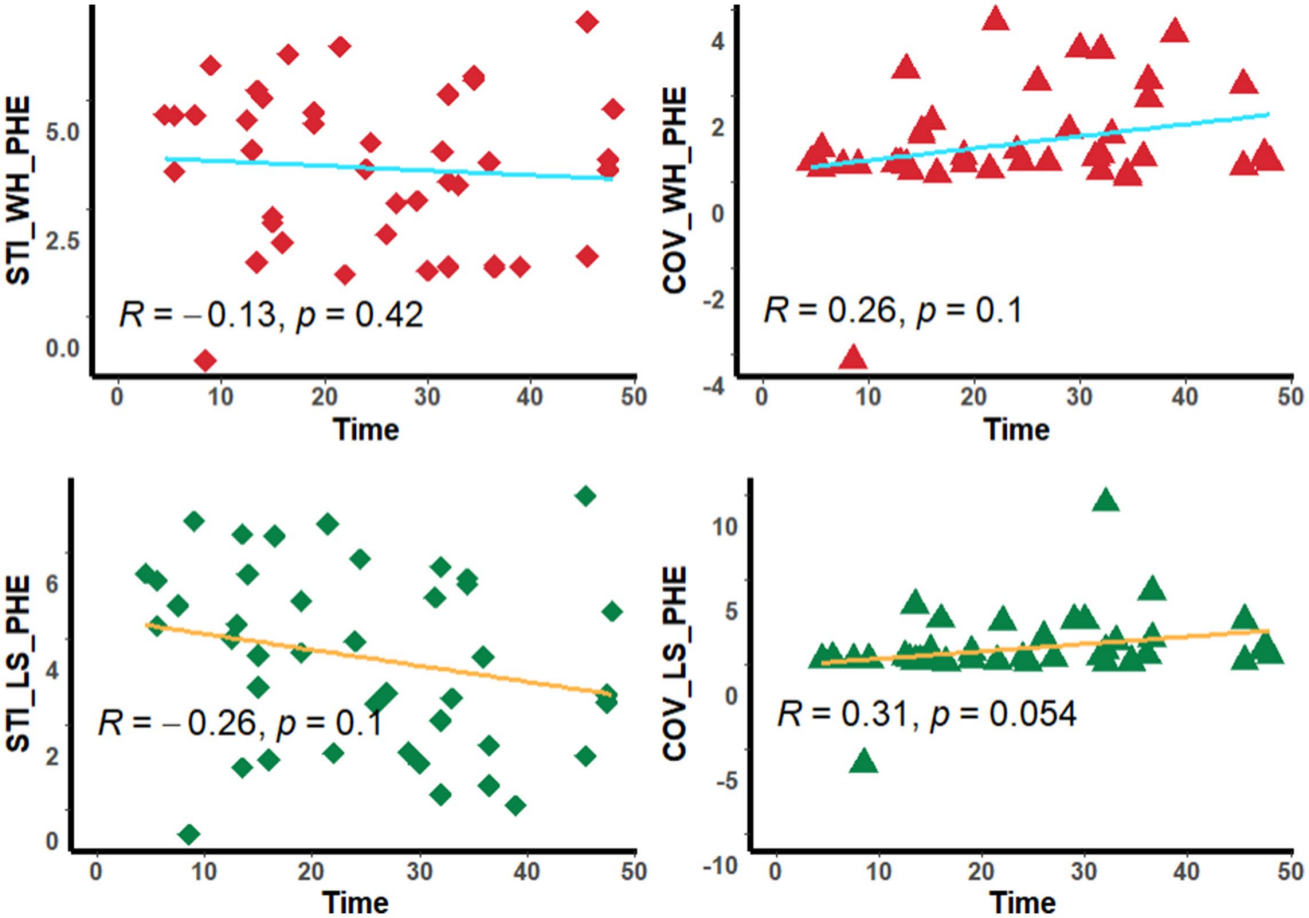
Scatter diagram. The diagram shows the corrlation between the stiffness index and time. STI_WH_PHE, mean stiffness index in the whole perihematomal edema region; STI_LS_PHE, mean stiffness index in the largest-slice perihematomal edema region; COV_WH_PHE, coefficient of variation of stiffness index in the whole perihematomal edema region; COV_LS_PHE, coefficient of variation of stiffness index in the largest-slice perihematomal edema region; Time, the time from onset to MRI scan.

Neither the mSTIs nor the COV in the whole PHE region and the largest-slice PHE region were significantly correlated with the time from onset or blood pressure (Figures 4, 5).

## 4 Discussion

Measuring the stiffness of perihematomal tissue may help us evaluate the development of ICH from a mechanical viewpoint. In this study, we built STI maps based on standard b-value DWI and evaluated the stiffness in the PHE regions. We found that the STI parameters in the PHE regions was closely correlated with the hematoma volume.

Stiffness is an important physical property of a substance. It is related to biological characteristics of tissues, and it can reflect the histopathological changes, as well as mechanical information in the tissues (22). In fact, brain has viscoelastic properties (with both viscosity and elasticity) (23), and exhibits heterogeneous stiffness due to the tissue heterogeneity (24–26). For ICH patients, the PHE region correlates with secondary brain injury (27), and undergoes pathological changes continuously over time (6), which may lead to a constant change in its stiffness.

Although magnetic resonance elastography (MRE) has a significant potential for application in brain diseases (28), it remains difficult to implement widely in current clinical practice. At present, the resolution of MRE is limited, and it should be treated with caution when comparing MRE results across studies as it may be affected by acquisition parameters, post-processing, and some other factors (29).

We know that DWI can reflect the diffusion of water molecules in tissues, and sDWI is one of the most commonly used sequences for MR imaging of brain diseases. In this study, we built a model for predicting virtual shear modulus using sDWI, and it showed good predictive performance. It is close to the result of a previous study that predicted liver shear modulus using sDWI parameters (21). This suggests that through sDWI examination, additional stiffness information may be obtained alongside diffusion restriction evaluation, making it easier to assess stiffness features of brain tissues affected by intracerebral hemorrhage or other diseases.

Our results showed that the STI parameters, including the mSTI and COV, in the largest-slice PHE region were significantly positively correlated with those in the whole PHE region. Also, the largest-slice mSTI and the COV showed a comparable correlation coefficient with the hematoma volume. These findings are very interesting. A previous study showed that the long axis of most hematomas is in the anterior–posterior direction, and the longitudinal axis type is unchanged in 90% of HE patients (30). This finding may indicate that the largest-slice stiffness in PHE regions have a potential value for evaluating the trend of hematoma development.

In this study, we found that the STI parameters in the PHE region are closely related to the hematoma volume; that is, the larger the hematoma volume is, the smaller the mSTI and the larger the COV of the STI. This might be because the increasing volume of the hematoma may cause pronounced edema (16), which may lead to a reduction in stiffness. The increasing COV value may indicate that the heterogeneity of the PHE region increases with increasing hematoma volumes.

It is known that time and blood pressure are important factors for ICH. However, our study revealed that neither the time from onset nor blood presure was significantly correlated with the STI parameters. This may be due to the complex pathological changes in the PHE regions over time (6), as well as the factors influencing the hematoma volume. This deserves further study.

An important application of our findings is to assess the possible direction of hematoma expansion using the STI map. Due to the heterogeneity of the tissue stiffness surrounding the hematoma, the hematoma may be more likely to expand to sites with less stiffness. The previous study revealed that HE in cerebral hemorrhage is asymmetrical in direction, and its shape becomes increasingly irregular with the expansion of the hematoma (30). Based on the STI map, the tissue stiffness around the hematoma can be measured to predict the direction of hematoma expansion, and timely treatment to avoid the involvement of important functional regions may be possible. Furthermore, as ICH is accompanied by damage to the surrounding brain tissue, therefore, stiffness evaluation by STI may be useful for quantitatively assessing the extent of brain injury.

There are several limitations in this study. First, the sample size of this study was small, and the results need to be further validated in a large multicenter sample. Second, we analyze only the correlations between the STI parameters and time from onset within 48 h. Subgroup analyses or a more detailed division of time point studies can be performed in the future to better understand the changes in the STI parameters with the development of ICH over time. Thirdly, although multistep preprocessing was performed, there may still be some effects near the hematoma when extracting the STI parameters from the PHE region. Fourthly, the blood pressure data used in this study were collected at the time of admission, and the data collected just before MRI examination may better reflect the relationship between blood pressure and stiffness around the hematoma. Fifthly, this study presents an evaluation of the STI of the tissue around the hematoma, which may reflect the relative magnitude of the stiffness, but there may be some differences from the true stiffness value.

## 5 Conclusion

In conclusion, we constructed STI maps using sDWI and evaluated the stiffness of PHE regions in ICH patients and showed that the STI parameters of PHE regions is closely correlated with the hematoma volume. This study may provide a way to evaluate ICH development from a mechanical point of view and also provide a target for ICH monitoring and treatment.

## Data Availability

The datasets presented in this article are not readily available because the raw data supporting the conclusions of this article are available from the corresponding author upon reasonable request. Requests to access the datasets should be directed to Fei Dong, dngfei@zju.edu.cn.

## Footnotes

^1^ https://fsl.fmrib.ox.ac.uk/fsl/fslwiki/

^2^ http://www.mrtrix.org/

^3^ https://www.nitrc.org/projects/captk/

^4^ http://www.itksnap.org

^5^ https://www.python.org/

^6^ http://www.r-project.org/
